# Coevolutionary feedback elevates constitutive immune defence: a protein network model

**DOI:** 10.1186/s12862-016-0667-3

**Published:** 2016-05-05

**Authors:** Tsukushi Kamiya, Leonardo Oña, Bregje Wertheim, G. Sander van Doorn

**Affiliations:** Groningen Institute for Evolutionary Life Sciences, University of Groningen, P.O. Box 11103, CC Groningen, 9700 The Netherlands; Department of Ecology and Evolutionary Biology, University of Toronto, 25 Willcocks Street, Toronto, Canada

**Keywords:** Optimal defence, Host-parasite coevolution, Constitutive immunity, Induced immunity, Immune network evolution, Individual-based simulation

## Abstract

**Background:**

Organisms have evolved a variety of defence mechanisms against natural enemies, which are typically used at the expense of other life history components. Induced defence mechanisms impose minor costs when pathogens are absent, but mounting an induced response can be time-consuming. Therefore, to ensure timely protection, organisms may partly rely on constitutive defence despite its sustained cost that renders it less economical. Existing theoretical models addressing the optimal combination of constitutive versus induced defence focus solely on host adaptation and ignore the fact that the efficacy of protection depends on genotype-specific host-parasite interactions. Here, we develop a signal-transduction network model inspired by the invertebrate innate immune system, in order to address the effect of parasite coevolution on the optimal combination of constitutive and induced defence.

**Results:**

Our analysis reveals that coevolution of parasites with specific immune components shifts the host’s optimal allocation from induced towards constitutive immunity. This effect is dependent upon whether receptors (for detection) or effectors (for elimination) are subjected to parasite counter-evolution. A parasite population subjected to a specific immune receptor can evolve heightened genetic diversity, which makes parasite detection more difficult for the hosts. We show that this coevolutionary feedback renders the induced immune response less efficient, forcing the hosts to invest more heavily in constitutive immunity. Parasites diversify to escape elimination by a specific effector too. However, this diversification does not alter the optimal balance between constitutive and induced defence: the reliance on constitutive defence is promoted by the receptor’s inability to detect, but not the effectors’ inability to eliminate parasites. If effectors are useless, hosts simply adapt to tolerate, rather than to invest in any defence against parasites. These contrasting results indicate that evolutionary feedback between host and parasite populations is a key factor shaping the selection regime for immune networks facing antagonistic coevolution.

**Conclusion:**

Parasite coevolution against specific immune defence alters the prediction of the optimal use of defence, and the effect of parasite coevolution varies between different immune components.

**Electronic supplementary material:**

The online version of this article (doi:10.1186/s12862-016-0667-3) contains supplementary material, which is available to authorized users.

## Background

Parasites threaten all living organisms. Immune defence is therefore crucial for providing protection from these enemies. However, defence is typically used at the expense of other fitness components and with the potential risk of self-damage. Therefore, hosts are faced with a challenge to optimise their defence deployment strategy so that it provides maximum protection while minimising costs [1, 2].

One important aspect of scheduling defence deployment is its temporal pattern of expression, which spans across the spectrum of constitutive to induced activation. A strategy is considered constitutive when an organism expresses a defensive phenotype regardless of external signals, that is, even in the absence of a threat. On the other hand, induced defence is only triggered upon detecting an enemy. Given that defence is costly, it is intuitively most economical to deploy defence only when a threat is encountered. However, physiological constraints often prevent an organism from mounting an immediate response, causing a delayed induced response of several hours to several days [3, 4]. Such a delay results in an unprotected period during which the risk of parasite-mediated harm is particularly high. Therefore, despite its sustained maintenance cost, constitutive defence can be adaptive for filling the potential period of unprotected exposure [5]. In nature, organisms deploy various combinations of constitutive and induced defence and a given effector molecule, whose function is to eliminate parasites, may be activated through both constitutive and inducible means. In the innate immune system of insects, for example, phenoloxidase (PO) is a defence effector molecule that is constitutively present, and can be further up-regulated upon induction through the proPhenoloxidase (PPO)-cascade [4].

Existing theoretical models of the optimal balance of constitutive and induced defence exclusively consider non-specific defence, assuming that hosts equally induce their defences, and that these defences are equally effective against all parasite genotypes (e.g. [5–8]). However, host-parasite interactions are characteristically genotype specific [9, 10]. In other words, it is the norm rather than the exception to find that hosts vary in their ability to resist parasites of different genotypes. For example, in vertebrate adaptive immunity, the major histocompatibility complex, MHC (also known as human leukocyte antigen, HLA) plays an important role in specific immune induction by binding to self and foreign peptides and displaying them on the cell surface for recognition by the T cells [10, 11]. High specificity of antigen presentation is made possible by a polymorphic lock-and-key mechanism: the MHC variable regions (lock) match the epitope, i.e., a fraction of the foreign antigen (key).

While specific defence was believed to be unique to the vertebrate adaptive immune system, this conventional belief has been challenged by studies of invertebrate immunity, which suggest neither adaptive immunity is restricted to vertebrates, nor specific defence is limited to adaptive immunity [9, 12–14]. For example, Carius et al. [15], using *Daphnia* and its bacterial parasite *Pasteuria ramosa*, showed different combinations of host and parasite genotypes create variation in infection success. The molecular mechanisms underlying the specificity in invertebrate innate immunity is not well understood to date [13]. Nonetheless, genetic studies in *Drosophila* indicate that there exists a considerable degree of specificity against parasites at both the receptor and effector level [16–18].

Specific defence is thought to be beneficial for the host, because high specificity may allow for more effective detection and elimination of certain parasite strains [13]. Conversely, rare parasite mutants that escape the specific defence would gain a large fitness advantage over common strains through negative frequency-dependent selection for rare advantageous alleles. Therefore, genotype-specific interactions are thought to maintain genetic diversity in both hosts and parasites, which is essential for antagonistic coevolution to continue [1]. Despite the ubiquity of genotype-specific host-parasite interactions, few theoretical studies have explicitly incorporated antagonistic coevolution into models of optimal defence [9, 19–21].

Here, we examine the effect of antagonistic parasite coevolution against the host receptor and effector on the optimal combination between constitutive and induced defence using a signal-transduction network model. A mechanistic perspective suggests that the immune system consists of a network of signal transduction cascades [4, 9, 13]. For example, in the innate immune system of *Drosophila*, once a non-self element (e.g. microbial ligand or parasitoid egg) is detected by a host protein (e.g. pattern recognition receptor), the system triggers a series of signalling pathways (e.g. *Toll* and *Imd* pathways) which lead to cellular responses such as phagocytosis and encapsulation, as well as the production of humoral effector molecules such as antimicrobial peptides and melanin [4]. In addition, signalling cascades exist that activate immune effectors constitutively (e.g. PPO-cascade) in the absence of parasite insults. Therefore, the success of an immune system depends not only on immune components that directly interact with parasites, but also on how those components are connected through a network of protein interactions [13].

While the network notion has long existed in the study of vertebrate adaptive immunity (i.e., immune network theory: [22, 23]), few evolutionary models have so far considered the immune system as a network (but see, [24]). Rather than predefining the direction and strength of interactions between immune components, the network approach allows them to emerge through in silico evolution. Another distinguishing feature of our analysis is that it addresses the effect of parasite coevolution on the optimal combination of constitutive and induced defence, complementing and extending previous studies that have investigated the optimal strategy for deploying constitutive and induced defences under evolutionary static conditions [5–8].

## Model

### Immune system as a signal transduction network

Following Soyer et al. [24–27], we employ an evolutionary approach to the analysis of signal transduction networks with the goal to evolve network interactions that achieve an optimal defence strategy. The innate immune system of invertebrates provides the inspiration for our model because of its relative simplicity and the wide array of constitutive to induced, and specific to non-specific defence mechanisms [1, 4].

**Fig. 1:**
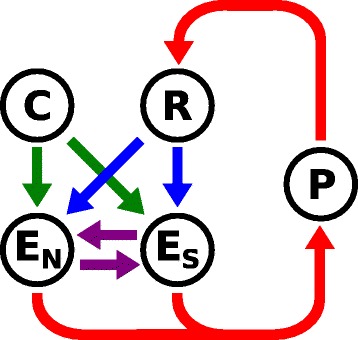
Immune network schematics. Immune network topology showing the interacting proteins (receptor, effector and constitutive proteins are denoted R, E, and C respectively) within a host individual and the parasite (P). The subscripts N and S with the effector refer to the non-specific and specific effector respectively

Rather than aiming for a mechanistically detailed description of invertebrate immunity for a particular model species, we take a conceptual, minimal modelling approach, and consider four proteins (which, in reality, may reflect entire modules of proteins and signal-transduction pathways): one recognition receptor (R), which is set either to be specific or non-specific, one constitutively active protein (C), and two resistance effectors (E_S_ and E_N_), one of which is specific and the other non-specific (Fig. 1). The recognition receptor may be activated upon detecting a parasite and resistance effectors may be triggered downstream of the signalling cascade to eliminate the parasite (Fig. 1, blue arrows). In addition, the constitutively active protein is capable of interacting with the effectors independent of external inputs (Fig. 1, green arrows). Lastly, effectors are able to regulate each other to allow such interactions as they are known in invertebrate immunity (Fig. 1, purple arrows) [13].

The above model design is highly simplified, but still flexible enough to allow for a wide range of specific to non-specific immune responses activated through a full spectrum of purely constitutive to purely induced stimulation. In addition it allows us to compare the evolution of immune components with varying degrees of specificity, and to consider the effect of specific defence both at the receptor and effector level. When host-parasite coevolution is taken into account, it is in the parasite’s interest to evade specific defense mechanisms, while the evolutionary interest of the host is the opposite. Therefore, the specific defence components coevolve antagonistically with the parasite, whereas the non-specific components are oblivious to coevolutionary pressures. We assume no difference in the intrinsic cost between specific and non-specific immune defence. However, a difference in the average efficacy between them can emerge in a population as a consequence of coevolution.

Following Soyer et al. [27], we assume that the total concentration of the proteins in our model is fixed, but each protein can occur in an active and inactive form. We keep track of the proportion of the active form of each protein, *y*_*i*_ (where *i*= R, C, E_S_ or E_N_, respectively, for the receptor, the constitutive protein and the two effector proteins). Biologically, protein activity could be mediated by various mechanisms, including phosphorylation and other reversible post-translational modification mechanisms.

If a protein *j* is connected to another protein *i* in the immune-network topology shown in Fig. 1, then the active form of protein *j* is allowed to mediate the activity of protein *i*. This interaction can be either activating (i.e., *j* catalyses the transition from the inactive to the active form of *i*) or inhibiting (*j* catalyses the reverse transition), depending on the sign of *μ*_*ij*_, the interaction coefficient for the two proteins. The protein interactions are directional; in particular, the activation or inhibition of protein *i* by protein *j* has no direct effect on the state of protein *j*.

Based on these assumptions, we formulate a system of ordinary differential equations to describe the dynamics of the protein activities in the absence of a parasite:

(1) $$  \frac{dy_{i}}{dt} = -\phi y_{i} + {\sum_{j}} c_{i\times j} \mu_{i\times j} y_{j} \times \left\{\begin{array}{ll} 1-y_{i} & \text{if }\mu_{i \times j}>0\,,\\ y_{i} & \text{if }\mu_{i \times j}<0\,. \end{array}\right.  $$

Here, *c*_*i*×*j*_ takes value one if protein *j* connects to *i* in Fig. 1, and zero otherwise ($\phantom {\dot {i}\!}c_{\mathrm {E}_{\mathrm {N}} \times \mathrm {R}} = c_{\mathrm {E}_{\mathrm {S}} \times \mathrm {R}} = c_{\mathrm {E}_{\mathrm {N}} \times \mathrm {C}} = c_{\mathrm {E}_{\mathrm {S}} \times \mathrm {C}} = c_{\mathrm {E}_{\mathrm {N}} \times \mathrm {E}_{\mathrm {S}}} = c_{\mathrm {E}_{\mathrm {S}} \times \mathrm {E}_{\mathrm {N}}} = 1$; all other *c*_*i*×*j*_=0). To ensure that protein activity equilibrates to zero in the absence of activating inputs, the equation contains a term that captures spontaneous deactivation of the active form of the protein. Spontaneous deactivation occurs at rate *ϕ*.

The interaction coefficients *μ*_*i*×*j*_ are considered to be evolving parameters. Their values are determined based on sequence matching between interacting proteins. To model this process, we assume that each of the proteins is characterised by three bitstring sequences (*S*_*N*_,*S*_*I*_ and *S*_*O*_) of length *L* (= 10): the first is a neutral reference sequence, the other two reflect the structure of, respectively, a protein input domain and an output domain, which form the interface of protein-protein interactions. The reference sequence has no phenotypic effects and merely accumulates neutral mutations, thus serving as a benchmark to detect adaptive evolution in the input and output domain (see Additional file 1 on the rate of molecular evolution).

The interaction coefficient *μ*_*i*×*j*_ between two host proteins is determined by the Hamming distance $H(S_{I}^{(i)}, S_{O}^{(j)})$ between the input domain of protein *i* and the output domain of protein *j*, according to the linear relationship:

(2) $$ \mu_{i \times j} = 1 - \frac{2 H\left(S_{I}^{(i)}, S_{O}^{(j)}\right)}{L} \,.  $$

Hence, the interaction is activating (*μ*_*i*×*j*_>0) when the number of matching bits between $S_{I}^{(i)}$ and $S_{O}^{(j)}$ is higher than *L*/2. Alternatively, the interaction is inhibiting (*μ*_*i*×*j*_<0) when the bitwise match is below 50 %. The determination of the interaction coefficients *μ*_*i*×*j*_ based on sequence matching makes it difficult for proteins to evolve strong interactions with many partners. By taking into account this evolutionary constraint, our model differs from previous evolutionary models of signal transduction networks [24–27], which allowed the interaction coefficients to evolve independently of each other.

### Host-parasite interactions and the degree of specificity

Equation (1) describes the dynamics of the immune network when the host is not challenged by a parasite. The same equation is used to model the induced response when the host interacts with a parasite, except that the summation over *j* then also includes a parasite protein P that interacts with the host receptor (*c*_R×P_=1; all other *c*_*i*×P_=0). The parasite protein is treated as a static inducer of the signalling cascade (Fig. 1, red arrow connecting P to R). That is, we do not consider the activity of P as a dynamic variable but substitute *y*_P_=1. The interaction coefficient *μ*_R×P_ is calculated in two different ways, depending on whether recognition is specific or not. In the scenario that the interaction between parasite and receptor is non-specific, *μ*_R×P_=0.2, irrespective of the sequence of the parasite protein. By contrast, if the interaction is specific, *μ*_R×P_=0 if the sequence match between the input domain of the receptor and the output domain of P, $1 - H(S_{I}^{(\mathrm {R})}, S_{O}^{(\mathrm {P})}) / L$, is less than 60 %, and *μ*_R×P_=1 otherwise. These assumptions are meant to reflect the common scenario that specific defence components are highly effective against a narrow range of enemies whereas non-specific components defend broadly with a lower efficacy.

The detection of the parasite (i.e., the activation of the receptor triggered by the presence of the parasite protein), can have subsequent downstream effects, such as on the activity of the two immune effectors. Their interaction with the parasite determines the probability of infection (Fig. 1, red arrows connecting E_S_ and E_N_ to P), which influences host survival as well as parasite reproductive success. The strength of the interaction between host effector *i* (= E_S_ or E_N_) and the parasite is measured by the interaction coefficients *ξ*_P×*i*_, which are determined by bitstring matching, in a way similar to how the specific and non-specific receptor interactions are modelled. To be exact, $\phantom {\dot {i}\!}\xi _{\mathrm {P} \times \mathrm {E}_{\mathrm {N}}} = 0.2$ for the non-specific effector, while the effectiveness of the specific immune effector depends on the Hamming distance between the output domain of E_S_ and the input domain of P: $\phantom {\dot {i}\!}\xi _{\mathrm {P} \times \mathrm {E}_{\mathrm {S}}} = 0$ if the sequence match $1 - H(S_{I}^{(\mathrm {P})}, S_{O}^{(\mathrm {E}_{\mathrm {S}})}) / L$ is less than 60 % and $\phantom {\dot {i}\!}\xi _{\mathrm {P} \times \mathrm {E}_{\mathrm {S}}} = 1$ otherwise.

The detection and the immune response against parasites frequently involve different parasite proteins. However, for clonally reproducing parasites this situation is equivalent to assuming a single parasite protein (as we do here) with separate output and input domains, $S_{O}^{(\mathrm {P})}$ and $S_{I}^{(\mathrm {P})}$, mediating interactions with, respectively, the host receptor and the immune effectors. Apart from the input and output domain, the parasite protein also contains a neutral bitstring of length *L*, which is used to quantify the rate of adaptive versus neutral sequence evolution (see Additional file 1 on the rate of molecular evolution).

### Immune deployment and fitness

The efficacy *E*_0_ and the fitness cost *C*_0_ of the constitutive immune defence of a host against a particular parasite are determined by the baseline activities of the two effectors:

(3) $$\begin{array}{*{20}l} E_{0} &= \xi_{\mathrm{P} \times \mathrm{E}_{\mathrm{S}}} \, y^{(0)}_{\mathrm{E}_{\mathrm{S}}} + \xi_{\mathrm{P} \times \mathrm{E}_{\mathrm{N}}} \, y^{(0)}_{\mathrm{E}_{\mathrm{N}}}\,, \end{array} $$

(4) $$\begin{array}{*{20}l} C_{0} &= \kappa \left(y^{(0)}_{\mathrm{E}_{\mathrm{S}}} + y^{(0)}_{\mathrm{E}_{\mathrm{N}}} \right)\, \end{array} $$

where $\phantom {\dot {i}\!}y^{(0)}_{\mathrm {E}_{\mathrm {S}}}$ and $\phantom {\dot {i}\!}y^{(0)}_{\mathrm {E}_{\mathrm {N}}}$ are equilibrium solutions of equation system (1), not including the interaction with the parasite. The parameter *κ* measures the marginal cost of expression of the specific and non-specific immune effector (i.e., the two responses are assumed to be equally costly).

A host may be challenged by a single randomly chosen parasite with an encounter probability that is varied as a parameter in the analysis. When a parasite is encountered, the activities of the immune effector can change as described in the previous section. The efficacy *E*^∗^ and cost *C*^∗^ of the induced immune defence are evaluated as:

(5) $$\begin{array}{*{20}l} E^{*} &= \xi_{\mathrm{P} \times \mathrm{E}_{\mathrm{S}}} y^{*}_{\mathrm{E}_{\mathrm{S}}} + \xi_{\mathrm{P} \times \mathrm{E}_{\mathrm{N}}} y^{*}_{\mathrm{E}_{\mathrm{N}}}, \end{array} $$

(6) $$\begin{array}{*{20}l} C^{*} &= \kappa \left(y^{*}_{\mathrm{E}_{\mathrm{S}}} + y^{*}_{\mathrm{E}_{\mathrm{N}}} \right)\, \end{array} $$

where $y^{*}_{\mathrm {E}_{\mathrm {S}}}$ and $y^{*}_{\mathrm {E}_{\mathrm {N}}}$ are the equilibrium solutions of equation system (1) including the interaction between the parasite and the host receptor.

The above definitions of efficacy and cost are intended to capture the relationship between the extent of immune response and the expression cost of the effector proteins. As a consequence of this relationship, hosts are faced with a trade-off between their ability to resist infection and minimising the cost of using immunity. Moreover, hosts need to balance the tradeoff between the costs and benefits of immunity under two different conditions: before and during infection.

Host survival before infection, *s*_0_=1−*C*_0_, depends only on the cost of constitutive defence, which is minimised when $\phantom {\dot {i}\!}y^{(0)}_{\mathrm {E}_{\mathrm {S}}} = y^{(0)}_{\mathrm {E}_{\mathrm {N}}} = 0$. However, during an infection, survival is also affected by the efficacy of defence. In particular, the probability of survival of an infected host, *s*^∗^, is calculated as:

(7) $$ \begin{aligned} s^{*} =&\, \left(\left(1 - C_{0}\right)\left(1 - \nu \, e^{-E_{0}} \right) \right)^{\delta}\\ &\times \left(\left(1 - C^{*}\right)\left(1 - \nu \, e^{-E^{*}} \right) \right)^{1-\delta} \,. \end{aligned}  $$

**Fig. 2:**
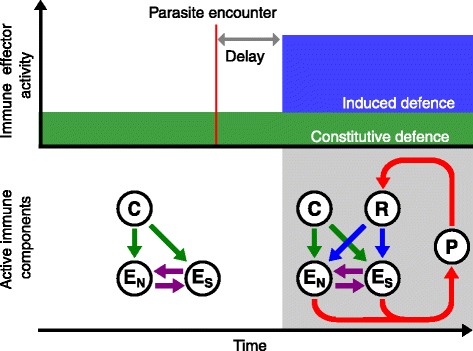
Immune effector activity and corresponding network architecture. A host expresses a fixed level of constitutive effector activity (green box; $y^{0}_{\mathrm {E_{S}}}$ and $y^{0}_{\mathrm {E_{N}}}$ in Eqs. 3 and 4) throughout its lifetime, enabled by the interactions between the constitutively active protein (C) and the effectors (E_S_ and E_N_). The induced effector activity (blue box) is defined as the difference between the effector activity following the parasite-receptor interaction ($y^{*}_{\mathrm {E_{S}}}$ and $y^{*}_{\mathrm {E_{N}}}$ in Eqs. 5 and 6) and the constitutive activity. This additional activity is triggered when a parasite (P) is detected by the receptor (R). A time delay is assumed between encountering a parasite and triggering the induced response. Both the cost and benefit of defence are realised as a function of the effector activity (Eqs. 3–6)

This expression takes into account the fact that the induced immune response is not mounted immediately when a parasite is encountered, so that the host is initially only equipped with constitutive immunity (Fig. 2). The induction delay, relative to the length of the infection, is determined by the parameter *δ*. During both phases of the infection (before and after the induced response is activated), survival depends on two factors that interact multiplicatively: the cost of immune expression, which is modelled as in the pre-infection phase, and parasite-induced mortality, which is proportional to the maximum virulence of the parasite, *ν*, and the probability of infection. The latter is a decreasing function of the efficacy of immune defence, *e*^−*E*^ (cf. [28]). Our model considers highly virulent parasites; up to 99 % of host fitness can be lost from an infection, should the immune system fail entirely. The overall fitness of the host is calculated as the product of the pre- and post-infection survival, i.e., *W*_host_=*s*_0_×*s*^∗^.

The fitness of a parasite is directly proportional to its probability of successful infection [28], which depends on the parasite’s ability to evade both the constitutive and the induced immune response:

(8) $$ W_{\text{parasite}} = e^{-\left(\delta E_{0} + (1-\delta) E^{*}\right)} \,.  $$

### Evolutionary simulation

Evolutionary simulations were initialised with randomly created host and parasite populations each consisting of 2000 genetically diverse haploid individuals. Generations were non-overlapping, population sizes were kept fixed (i.e., the densities of both host and parasite populations were assumed to be regulated by external mechanisms), and both host and parasite populations were well mixed. Host individuals produced offspring by sexual reproduction. The parents of each offspring were sampled from the population using a fitness-weighted lottery scheme with replacement. Recombination was allowed to occur between, but not within, proteins. Parasites were assumed to reproduce asexually, based on a fitness-weighted lottery with replacement. Genetic variation in the host and parasite populations was introduced by point mutations in the protein bitstring sequences, which occurred at a rate of 1 % per individual per generation. All results presented are the average over 20 simulations, each run for 10000 generations with a burn-in period of 5000 generations to allow for the build-up of genetic variation and to eliminate initial effects. Simulation programmes were written in C^++^ (see Additional file 2) and statistical analyses were carried out in R 3.0.1.

## Results

Our analyses focus on three different scenarios. We first consider non-specific detection and focus on the role of the non-specific effector in the elimination of the parasite, thus establishing a baseline for the two other scenarios, which consider specific defence components at the downstream and upstream end of the immune signalling cascade. The second scenario is similar to the first one, except that we concentrate on the contribution of the specific effector towards immunity. In the third scenario, we study the effect of specific detection, by configuring the immune network with a specific receptor. For this case, we present only the activity of the non-specific effector, since the effects of specific detection and specific elimination were found to be additive. Supplementary results concerning the rate of evolution and the interaction patterns between proteins that emerged through evolution are provided in the Additional files 1 and 3.

### 1) No parasite coevolution: non-specific detection and non-specific elimination

Non-specific defence is triggered by generic parasite signals [10]. In our model, non-specific immune components interact with an encountered parasite regardless of host-parasite genotype-matching, such that non-specific defence is unaffected by antagonistic parasite coevolution. Despite adopting a different modelling approach, the non-specific components of our model mirror predictions from previous models examining the optimal combination of constitutive versus induced defence: we observe the evolution of networks that implement a mixed immune strategy of constitutive and induced defence. Moreover, the optimal balance of the two defence strategies is found to depend on the probability of parasite encounter, the cost of immune defence and the induction delay.

**Fig. 3:**
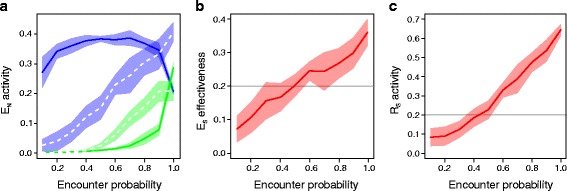
Effect of encounter probability. **a** Non-specific effector (E_N_) activity against the probability of parasite encounters. The green lines indicate the level of constitutive expression while the blue lines indicate the induced expression level. The results of the non-specific detection case (Scenario 1) are the solid lines and those of the specific detection case (Scenario 3) are shown by the dashed lines. **b** Specific effector efficacy (E_S_) and (**c**) specific receptor (R_S_) activity against the probability of parasite encounters. The horizontal grey line is the non-specific receptor activity and non-specific effector efficacy in (**b**) and (**c**) respectively. The mean and standard error bands across 20 replicate simulations are shown. The cost of immunity and induction delay are both set low (i.e., 0.2); the result is qualitatively identical for any combination of the range in cost of immunity and induction delay explored (0.2 to 0.8; results not shown). Fixed parameter values are as follows: sequence length = 10, spontaneous deactivation rate = 0.3, host and parasite mutation rate = 0.01, host and parasite population size = 2000 and maximum virulence = 0.99

**Fig. 4:**
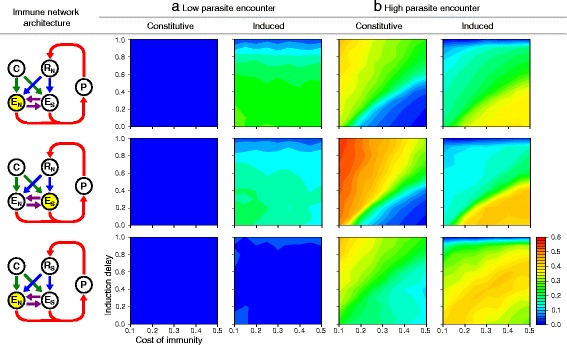
Effector activity. Constitutive and induced effector activity under (**a**) low and (**b**) high parasite encounter probability (0.2 and 1.0, respectively) and three different host-parasite coevolutionary scenarios: no parasite coevolution (Scenario 1: non-specific detection and elimination; top row), parasite coevolution against the host effector (Scenario 2: non-specific detection and specific elimination; middle row), and parasite coevolution against the host receptor (Scenario 3: specific detection and non-specific elimination; bottom row). The effector for which the activity level is shown is highlighted in yellow. Fixed parameter values are as given in Fig. 3 caption

In particular, our model predicts that the relative amount of constitutive to induced defence increases with the probability of parasite encounters (Fig. 3a; solid lines), in agreement with the existing theoretical literature [5–8]. While effector expression is wholly induced through the receptor when the encounter probability is low, the induced expression decreases as the effector starts to be activated by the constitutively active pathway. The lack of constitutive defence at low rates of parasite encounter (Fig. 4a; top row) is explained by the fact that the cost outweighs the benefit of pre-emptive allocation into defence if threats are rarely encountered. When the encounter probability is high (Fig. 4b; top row), mixed strategies of constitutive and induced defence evolve. The optimal balance between the two depends on the physiological constraints of the two activation strategies (Fig. 4b; top row). When the cost of using one’s immune system is low and induction delay is high, defence relies on constitutive activation. In contrast, induced activation is favoured over pre-emptive investment when it is readily deployable and the cost of immunity is so high that it is cost ineffective to maintain defence constitutively.

### 2) The effects of parasite coevolution against host effector: non-specific detection and specific elimination

In our second scenario, we focus on the specific effector, which allows the host genotype-specific elimination of parasites and opens up the possibility for coevolution between the specific effector and the parasite input sequence. When the encounter probability is low, there is very little constitutive activation of the specific effector. This is partially because induced activation is a more cost-effective option as seen above (Fig. 4a; middle row). Here, constitutive activation is further hampered by the fact that the specific effector is under weak selection, since it rarely encounters a parasite. As a result, the host does not evolve fast enough to maintain an effective specific immune response in the coevolutionary race with the parasite. As the parasite encounter rate increases, the specific effector is used more frequently, creating the opportunity for selection to maintain a cost-effective specific immune response (Figs. 3b and 4b).

Hosts enjoy a greater benefit of immunity by allocating resources to an effective effector. Therefore, when the encounter probability is high, the specific effector is activated more than the non-specific effector, both through the constitutive and the induced activation pathway. This finding mirrors the insights of Jokela et al. [19] that the magnitude of immune responses should reflect the effectiveness of the immune system, which is subjected to parasite coevolution. Nonetheless, the balance of constitutive versus induced activation of the specific effector is similar to that of the non-specific effector.

### 3) The effects of parasite coevolution against host receptor: specific detection and non-specific elimination

In the third scenario, we consider that the immune system is induced by a specific receptor and we concentrate once more on the activity of the non-specific effector. This set-up allows for genotype-specific detection of parasites and opens up the possibility for coevolution between the specific receptor and the parasite output sequence. Detection of a parasite by the host receptor is the first step of any induced immune response. Since the chance of successful infection increases dramatically if a parasite manages to evade detection, host immune receptors experience ample antagonistic coevolutionary pressures.

When the encounter probability is low, constitutive defence is uneconomical, forcing the defence system to rely upon induction, as we saw in the previous sections. However, when the host hardly ever encounters a parasite, the host receptors are unable to evolve fast enough to maintain the close sequence match with the parasite that is required to enable detection (Fig. 3c). Therefore, the specific receptor is rarely activated to induce effector expression (Fig. 4a; bottom row). This finding indicates that hosts would adapt to tolerate rather than attempt to resist infection if parasite detection is unsuccessful (even despite having the possibility of using constitutive defence), corroborating the finding by Jokela et al. [19] that hosts should tolerate rather than resist infection when defence is ineffective.

Though constitutive expression increases, as before, when the encounter rate increases (Fig. 3a; dashed lines), the same happens with the induced expression, because parasite detection by the specific receptor improves as it is used more regularly and thereby exposed to selection more often (Fig. 3c). The resulting pattern is in contrast with the non-specific receptor scenario and other host-centric models [5–8] which predict that the relative amount of constitutive to induced defence increases with increasing parasite encounter probability (Fig. 3).

**Fig. 5:**
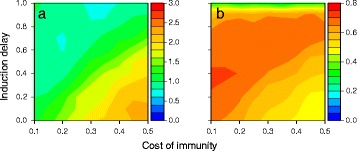
Parasite coevolution and receptor activity. **a** Parasite diversity measured as the pairwise genetic distance of parasites coevolving against hosts with a specific receptor (R_S_), and (**b**) the corresponding host R_S_ activity, which equates to the detection rate of encountered parasites. Fixed parameter values are as given in Fig. 3 caption

A second counterintuitive result at high encounter probability is that increased induction delay, which diminishes the benefit of induction, elevates the level of defence induced by the specific receptor (Fig. 4b; bottom row). This is explained by the fact that the probability of infection depends to a large degree on receptor performance when protection relies predominantly upon induced defence (i.e., when the cost of immunity is great and induced delay is short). Consequently, parasites are under strong positive selection pressure to evade detection when induction is most beneficial to the host (Additional file 1), resulting in the evolutionary diversification of the parasite population (Fig. 5a). Parasite diversification prevents hosts from adequately responding to all different parasite genotypes, reducing the average receptor and, hence, effector activity. To compensate for the resulting loss of protection, hosts invest more heavily in constitutive defence (Fig. 4b; bottom row) by evolving an elevated level of constitutive activation (Additional file 3). On the other hand, when host fitness depends less strongly on the induced immune response (i.e., when induction delay is larger), the selection pressure on parasites to escape detection is less severe, reducing its ability to support the maintenance of genetic variation (Fig. 5a). Therefore, hosts are better able to detect the parasite, leading to a higher average level of activation of the specific receptor (Fig. 5b) and, consequently, an elevated induced effector response.

**Fig. 6:**
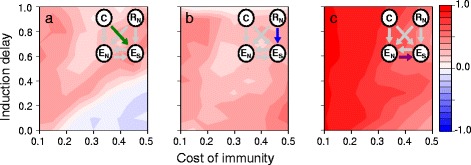
Specific effector activation. Evolved protein interactions under high encounter probability (=1.0). The protein network diagram depicts interacting proteins. Arrow colours refer to the constitutive (**a**, *green*) and induced (**b**, *blue*) interactions towards the specific effector (E_S_), and the regulatory input from the non-specific (E_N_) to the specific (E_S_) effector (**c**, *purple*). Fixed parameter values are as given in Fig. 3 caption. See the Additional file 3 for the full list of evolved protein interactions

By allowing the interactions among host immune proteins to evolve freely, we also gain some insight into how the architecture of an immune signal transduction network may be shaped by host-parasite coevolution. Our simple network shows that the specific effector is activated, mostly indirectly, through the non-specific effector (Fig. 6): the indirect activation from the non-specific effector (Fig. 6c) is much stronger than the direct input from either the constitutive (Fig. 6a) or the receptor protein (Fig. 6b). This indirect activation is due to a trade-off in protein interactions imposed by sequence matching so that the network is genetically constrained from optimising all parts of the network simultaneously. The result indicates that in order to highly express a specific effector, the non-specific effector must also be highly activated, suggesting that the non-specific effector provides the baseline defence and the specific effector plays a supplementary role.

## Discussion

The present study was motivated by calls to incorporate parasite evolution into the study of host adaptation [9, 19, 29]. Most theoretical studies of the evolution of host defence strategies are host-centric [19]; meaning that hosts evolve to optimise their strategy while parasites are unable to counter-adapt to changing host strategies. We here demonstrate that parasite coevolution against specific immune defence alters the prediction of optimal deployment strategies based on such models, and that the effect of parasite coevolution varies between different immune components. Coevolution at the effector level does not alter the optimal balance between constitutive and induced defence qualitatively. Yet, investment into defence could exceed what is expected from the non-specific effectors if the specific effector is more potent. On the other hand, coevolution at the receptor level obscures the prediction of host-centric models [5–8], that the relative investment into constitutive over induced defence increases with parasite encounter probability. The cause of the discrepancy between host-centric models and the current coevolutionary analysis is that the balance of control over the outcome of antagonistic coevolution shifts between host and parasite depending on ecological conditions and the role of physiological constraints.

Using a within-host model, Hamilton et al. [7] show that variability in parasite growth rates selects for the host to adapt a combination of constitutive and induced defences because uncertainty favours induced over constitutive defence. Hamilton et al. [7] implemented parasite variability as a fixed parameter. Here, we show that specific host defence selects for adaptive diversification of the parasite population, as one would expect from models of host-parasite coevolution (e.g. [30–32]). This evolutionary feedback between host and parasite populations causes parasite diversity to correlate negatively with the strength of immune induction (Fig. 5), complicating the interpretation of the role of parasite variability in favouring induced defence.

In our model, hosts and parasites have symmetrical potential for adaptation; they are equal in population size, mutation rate and generation time. However, parasites are often thought to have an evolutionary edge over their hosts for having a larger population size and a shorter generation time. When parasites undergo a faster rate of evolution than their host, as is commonly believed [1], the effect of parasite diversity on the specific receptor activity is expected to be even greater than shown in the present study. Therefore, predictions from host-centric models that do not consider parasite coadaptation are likely to be particularly misleading for hosts facing fast evolving parasites, such as viruses.

Our present model made several simplifying assumptions about parasite infection dynamics. First, constant host and parasite population sizes were assumed, as is commonly done in theoretical studies of genotype-specific host-parasite interactions. Including the population dynamics of host and parasite populations is an important ingredient to better capture the eco-evolutionary feedback between the interacting species. Second, a single host was not allowed to be challenged by multiple parasites, which excluded competition between parasite genotypes as a source of selection. Third, within-host dynamics of parasite growth and immunological variables were ignored because our model focused on the effects of genotype-specific interactions from a population perspective. Future studies may benefit from considering within-host dynamics because such an approach may open possibilities for empirical validation of a model with the available data on temporal expression patterns of immune proteins [33–36]. Finally, since our model only makes rather generic assumptions about the architecture of the immune system, the results may apply to a broad class of species that can alter their relative investment in constitutive versus induced defence.

## Conclusion

The present analysis indicates that interactions of multiple immune components and evolutionary feedbacks shape the evolution of host defence strategies. To gain a more complete understanding of immune systems subjected to coevolution, crucial empirical challenges await in, first, elucidating mechanisms and functions of basic building blocks, and second, characterising molecular interactions among those components. Invertebrate immune systems, especially that of *Drosophila*, for which a wealth of molecular information is available, present obvious candidates to pursue such challenges in the future [37]. A promising avenue in this area of research is to integrate empirical work on well-characterised model systems with theoretical models of immune network evolution to generate testable hypotheses on the functional roles of building blocks and pathways in immune systems consisting of a complex network of molecular interactions [1].

## Additional files

Additional file 1 The rate of molecular evolution. The rate of evolution for each protein including the parasite presented as the log(Ka/Ks) shown for the parameter combinations presented in Fig. 4. (PDF 176 kb)

Additional file 2 C^++^ source code of the simulation programme. (ZIP 25.9 kb)

Additional file 3 Evolved protein interactions. Protein interactions emerged through evolution shown for the parameter combinations presented in Fig. 4. (PDF 142 kb)
