# Supplementary material for: Coevolutionary feedback elevates constitutive immune defence: a protein network model
Source: BMC Evol Biol. 2016 May 5;16:92. doi: 10.1186/s12862-016-0667-3 (PMC4858902; doi:10.1186/s12862-016-0667-3)
Supplement: Additional file 1 — The rate of molecular evolution. The rate of evolution for each protein including the parasite presented as the log(Ka/Ks) shown for the parameter combinations presented in Fig. 4. (PDF 176 kb) [file 12862_2016_667_MOESM1_ESM.pdf]

Additional File 1

The rate of molecular evolution

The rate of evolution for a protein domain, K, i.e. the overall change of a given protein domain between two census events, is calculated as the sum of the proportional difference at homologous loci between two censuses:

$$K = \sum_{i=1}^{\eta} = a_i(1-b_i) + b_i(1-a_i)$$

where η is the size of sequence domains, a_i and b_i are the proportion of bitstring value 1 at time t and $t + \Delta t$ respectively. Therefore $a_i(1 - b_i)$ denotes the proportion of bitstrings that change from 1 to 0 while $b_i(1 - a_i)$ denotes the proportion of bitstrings that change from 0 to 1 between two census events, summing to K, which represents the total change of a protein domain.

A consensus sequence is created at each census of the population by calculating the average proportion of 1 at each locus of each protein domain. The time interval between two censuses (Δt) is determined by the amount of neutral mutations accumulated since t: the cumulative neutral mutations are quantified every five generations and when the value, K, for the neutral domain sequences sampled at t and $t + \Delta t$ exceeds a threshold (i.e. 0.5), Δt is taken as the sampling interval.

As described in the main text, a protein consists of three sequence domains: input, output and neutral reference. Following the convention of population genetics, we denote the rate of evolution, K, as K_s and K_a for the synonymous neutral domain and non-synonymous input and output domains respectively.

The results of the rate of evolution are shown in the Additional File Figure 1. Overall, the signature of selection is consistently positive for sequences involved in coevolutionary interactions. For other sequences, selection is predominantly stabilising indicating that selection operates to maintain the protein interactions within the immune system once the system has had enough time to evolve.

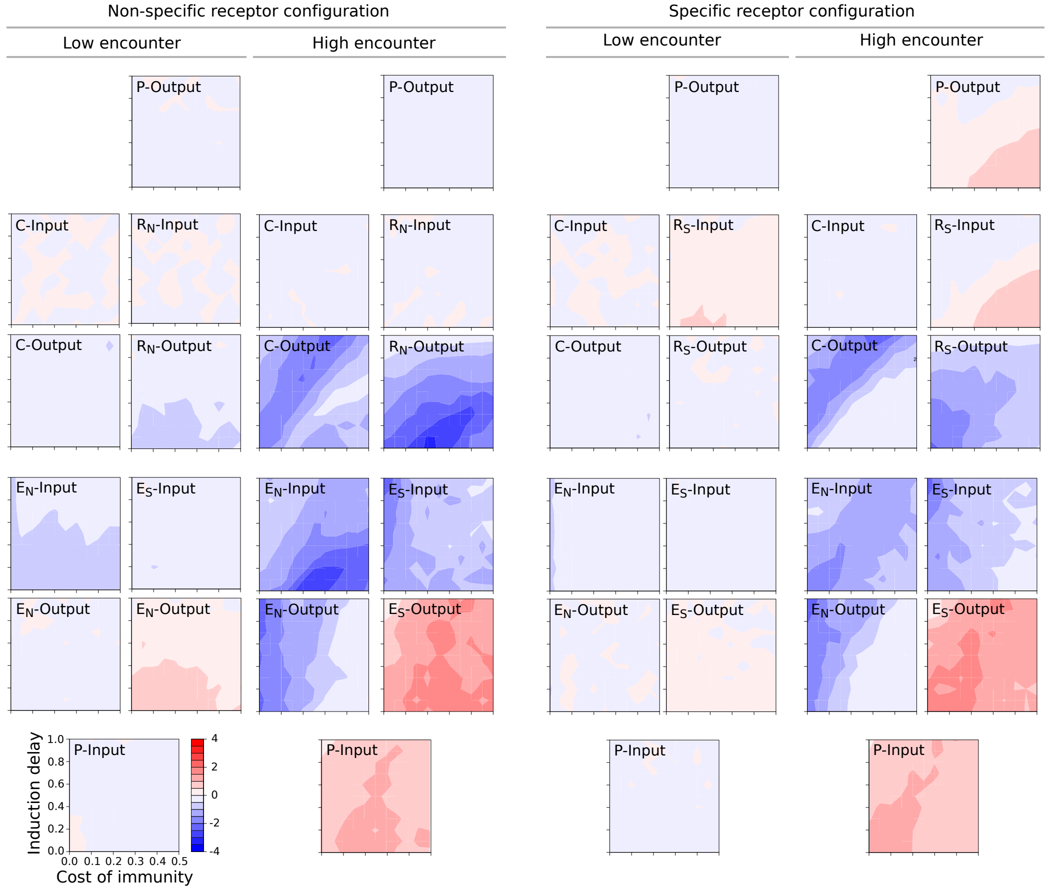

Figure 1: The rate of evolution for each protein including the parasite (P) presented as the $\log(K_a/K_s)$ shown for the parameter combinations presented in Figure 4. Positive values in red show a sign of positive selection while negative values in blue are indicative of stabilising selection. Each host consists of four proteins: a constitutively active protein (C), a receptor which may be either non-specific (R_N) or specific (R_S) and two effectors, one of which is non-specific (E_N) and the other specific (E_S). Each protein consists of two functional sequence domains under selection: input and output. The results are presented for the combination of non-specific and specific receptor configurations and low (=0.2) and high (=1.0) parasite encounter probabilities.
