# Supplementary material for: Coevolutionary feedback elevates constitutive immune defence: a protein network model
Source: BMC Evol Biol. 2016 May 5;16:92. doi: 10.1186/s12862-016-0667-3 (PMC4858902; doi:10.1186/s12862-016-0667-3)
Supplement: Additional file 3 — Evolved protein interactions. Protein interactions emerged through evolution shown for the parameter combinations presented in Fig. 4. (PDF 142 kb) [file 12862_2016_667_MOESM3_ESM.pdf]

Additional File 3

Evolved protein interactions

Our network approach allowed the interactions between immune proteins to emerge through *in silico* evolution and the evolved interaction patterns are shown in the Additional File 3 Figure 1.

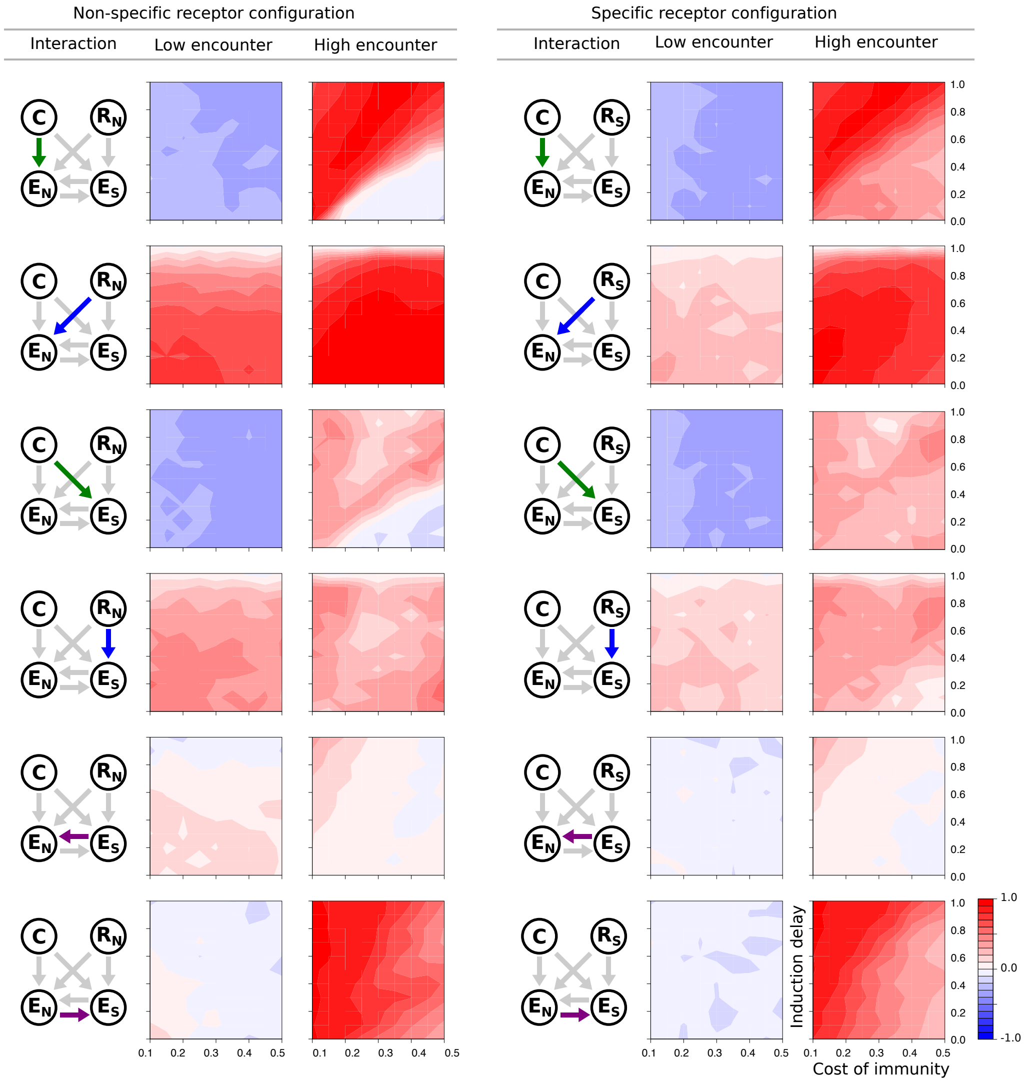

Figure 1: Six protein interactions that emerged through evolution are presented for the parameter combinations used in Figure 4 in the main text. The arrows indicate the focal interaction. The arrows colours reflect the type of each interaction, which are: constitutive to effector interaction (green), receptor to effector interaction (blue) and effector regulations (purple). Positive values in red show activating interactions while negative values in blue are inhibiting interactions. Each host consists of four proteins: a constitutively active protein (C), a receptor which may be either non-specific (R_N) or specific (R_S) and two effectors, one of which is non-specific (E_N) and the other specific (E_S). The results are presented for the combination of non-specific and specific receptor configurations and low (=0.2) and high (=1.0) parasite encounter probabilities.
